# Viral pneumonia in adults and older children in sub-Saharan Africa — epidemiology, aetiology, diagnosis and management

**DOI:** 10.15172/pneu.2014.5/446

**Published:** 2014-12-01

**Authors:** Antonia Ho

**Affiliations:** 14 Malawi Liverpool Wellcome Trust Clinical Research Programme, Chichiri, Blantyre 3, Malawi; 24 Institute of Infection and Global Health, University of Liverpool, Liverpool, UK

**Keywords:** viral pneumonia, viral respiratory tract infections, adult, older children, Africa, sub-Saharan Africa

## Abstract

Community-acquired pneumonia causes substantial morbidity and mortality in sub-Saharan Africa with an estimated 131 million new cases each year. Viruses — such as influenza virus, respiratory syncytial virus and parainfluenza virus — are now recognised as important causes of respiratory disease in older children and adults in the developed world following the emergence of sensitive molecular diagnostic tests, recent severe viral epidemics, and the discovery of novel viruses. Few studies have comprehensively evaluated the viral aetiology of adult pneumonia in Africa, but it is likely to differ from Western settings due to varying seasonality and the high proportion of patients with immunosuppression and co-morbidities. Emerging data suggest a high prevalence of viral pathogens, as well as multiple viral and viral/bacterial infections in African adults with pneumonia. However, the interpretation of positive results from highly sensitive polymerase chain reaction tests can be challenging. Therapeutic and preventative options against viral respiratory infections are currently limited in the African setting. This review summarises the current state of the epidemiology, aetiology, diagnosis and management of viral pneumonia in sub-Saharan Africa.

## 1. Introduction

Community-acquired pneumonia (CAP) is a leading cause of morbidity and mortality in all age groups worldwide (1). The African continent carries a substantial burden — around 30% of the estimated 430 million episodes of lower respiratory tract infections (LRTI) each year (1). Bacteria, for example, *Streptococcus pneumoniae*, are the principal causative agents in adult pneumonia (2, 3). However, the availability of sensitive molecular tests has resulted in the increasing recognition of viruses as major contributors (4, 5). Moreover, the emergence of avian influenza A(H5N1) virus and 2009 pandemic influenza A(H1N1) virus, along with the accelerated discovery of other novel respiratory viruses (e.g., human metapneumovirus [hMPV], bocavirus, coronaviruses NL63 and HKU1, Middle East Respiratory Syndrome [MERS]), against a backdrop of a growing population of immunocompromised individuals has catalysed research attention on severe viral respiratory infections in adults.

Available data on the contribution of viruses to adult CAP comes mainly from studies in developed settings (4, 5). Viral causes of pneumonia in African adults are less well characterised, largely due to the lack of access to appropriate diagnostics. The epidemiology, aetiological agents, seasonality, and age distribution of patients are likely to differ substantially from the developed setting for a myriad of reasons. Firstly, respiratory viruses typically follow seasonal patterns. In contrast to the distinct winter epidemics as seen in temperate regions, available influenza data indicate year round transmission in most African regions (6). Secondly, high prevalence of human immunodeficiency virus (HIV) infection and other comorbidities (e.g., tuberculosis [TB]) are likely to impact on the epidemiology and microbial aetiology of adult viral respiratory presentations (7). Several pneumonia aetiology studies in Africa have described a preponderance of younger adults (aged 15–45 years) (8, 9). Additionally, LRTI with herpes viruses, such as cytomegalovirus (CMV), herpes simplex virus (HSV) and varicella zoster virus (VZV), are common in adults in Western settings (10) and children in sub-Saharan Africa (11) with advanced immunosuppression. Moreover, immunocompromised patients may have more severe presentations. Emerging data from South Africa describes higher risk of death in HIV-infected adults with influenza infection (12). Lastly, many developed settings recommend targeted (13) or universal (14) seasonal influenza vaccination, but few African countries have national influenza vaccination programmes (15).

This paper reviews the current knowledge on the epidemiology and aetiology of viral pneumonia in adults and older children in sub-Saharan Africa, and evaluates the available options for diagnosis, prevention and management, and viral respiratory pathogens in this resource-poor setting.

## 2. Definitions and search strategy

The absence of a standardised case definition has been a longstanding problem of pneumonia aetiology studies. Most studies undertaken in developed settings have focused on radiologically-confirmed pneumonia (i.e. the presence of new infiltrates on chest radiograph [x-ray] with clinical symptoms suggestive of LRTI) (16–25). Given the inconsistent availability of chest radiographs in African health settings (26), a broader definition of pneumonia was used to include clinical evidence of LRTI with or without confirmed chest radiograph changes.

A literature search of original research articles using the PubMed database from inception through 31 May 2014 was conducted. The following PubMed search strategy was used: (“acute respiratory infection”[All Fields] OR “acute lower respiratory tract infection” [All Fields] OR “severe acute respiratory infection”[All Fields] OR “pneumonia”[All Fields] OR “pneumonia”[ MeSH Terms]) AND (“Africa” [MeSH Terms] OR “Africa” [All fields]).

**Figure 1:**
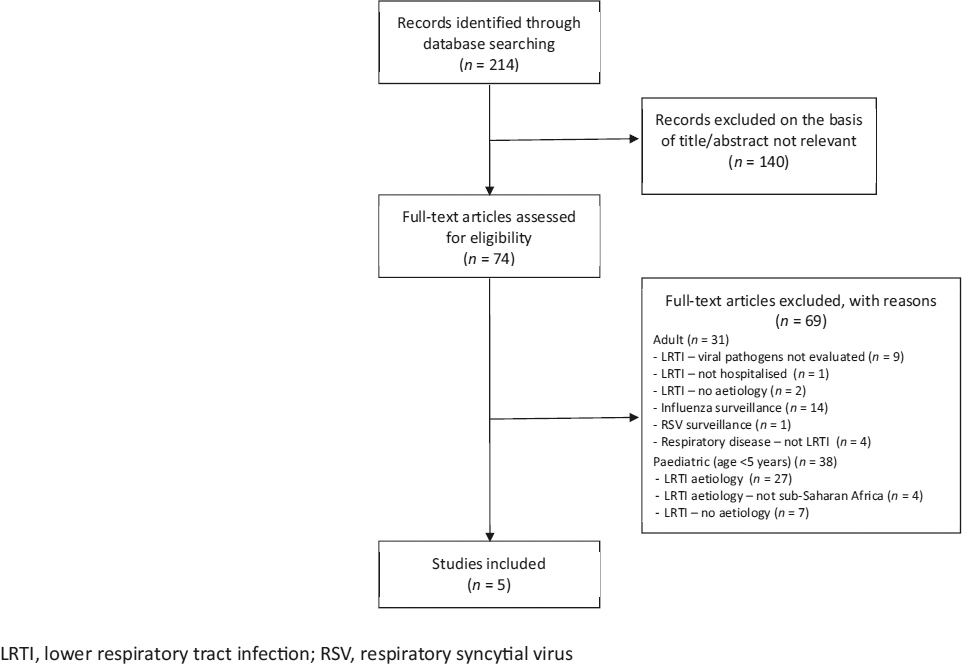
Flow chart of identification and selection of studies that have evaluated viral aetiology of pneumonia in adults and older children in sub-Saharan Africa

The following inclusion criteria were used: (i) study population of patients aged >5 years of age; (ii) study population of patients with radiologically-confirmed pneumonia or clinical LRTI; (iii) study population of patients that have been hospitalised; and (iv) study in any language. Since the purpose of this review was to describe the viral causative pathogens of African adults and older children presenting with pneumonia, studies that only examined for influenza or bacterial pathogens were excluded (Figure 1).

## 3. Viral epidemiology and aetiology in sub-Saharan Africa

Few studies have comprehensively examined the microbial aetiology of pneumonia in Africa; even fewer have included viral diagnostics. A growing number of acute respiratory infection (ARI) surveillance studies in sub-Saharan Africa, with particular interest in influenza virus (27–41) and respiratory syncytial virus (RSV) (42), have been published since the emergence of pandemic influenza A(H1N1) virus in 2009, reflecting the rapid expansion in surveillance capacity in the region (43). However, most were excluded from this review as they focused on a single virus and did not fulfill the inclusion criteria of patients hospitalised with LRTI. We identified 5 studies in African individuals aged 5 years and over with hospitalised LRTI in whom viral aetiology was evaluated (8, 39, 41, 44, 45) (Tables 1 and 2). There was substantial heterogeneity in the study populations, clinical case definitions of “pneumonia” (in terms of types and duration of clinical symptoms, and requirement for chest radiograph changes), and clinical specimens obtained for viral testing (Table 1). Three of the 5 studies did not include whole years of observation (44, 46), which may over- or underestimate the burden of viral infections depending on the study period included. Two studies conducted prior to 2000 used viral culture and serology (8, 44). The others utilised polymerase chain reaction (PCR)-based methods. All studies tested for influenza A and B viruses, adenovirus and RSV, and most also examined for parainfluenza virus (PIV) and enteroviruses. More recent studies have encompassed testing for rhinovirus (39, 41, 45), hMPV (39, 41, 45), coronaviruses (45) and bocavirus (45). Of note, none tested for CMV. HIV prevalence was high (49–94%) among the 3 studies that reported serostatus (8, 39, 45).

**Table 1:** Characteristics of studies on viral aetiology in hospitalised adults and older children with pneumonia or clinical lower respiratory tract infection in sub-Saharan Africa.

Study, year [ ] Reference	Country	Study period	Setting	Study population	“Pneumonia” definition	Specimen	Viral diagnostic test
Joosting et al., 1979 [44]	South Africa	May 1966–Apr 1972	Hospital-based observational study	Black miners	Acute respiratory disease (definition not stated)	Throat swabs & serum	Viral culture & serology (HAI or complement fixation)
Scott et al., 2000 [8]	Kenya	Mar 1994–May 1996	Hospital-based observational study	>15 years	Pneumonia (>2 symptoms of fever, cough, sputum, chest pain, SOB, or haemoptysis; symptoms <14 days; consolidation on CXR)	Serum	Complement fixation
Hartung et al., 2011 [45]	Malawi	Feb 2006–Sept 2006	Hospital-based observational study	>18 years	Pneumonia (>1 symptom of cough, sputum, chest pain, SOB, chest pain or haemoptysis; CXR changes) + admission to HDU	BAL fluid	rRT-PCR
Feikin et al., 2012 [39]	Kenya	Mar 2007–Feb 2010	Population-based surveillance	>5 years	ARI (cough or difficulty breathing or chest pain and temperature >38.0 °C or oxygen saturation <90% or hospitalisation)	NP & OP swabs	rRT-PCR
Pretorius et al., 2012 [41]	South Africa	Feb 2009–Dec 2010	Hospital-based surveillance (6 hospitals)	>5 years	SARI (fever; cough or sore throat; shortness of breath or difficulty breathing; symptoms <7 days)	NP & OP swabs	rRT-PCR

HAI, haemaglutination inhibition assay; SOB, shortness of breath; CXR, chest radiograph (x-ray); HDU, high dependency unit; BAL, bronchoalveolar lavage; rRT-PCR, real-time reverse transcriptase polymerase chain reaction; ARI, acute respiratory infection; NP, nasopharyngeal; OP, oropharyngeal; SARI, severe acute respiratory infection.

**Table 2:** Results of studies on viral aetiology in hospitalised adults and older children with acute lower respiratory tract infection or pneumonia in sub-Saharan Africa

Study, year [ ] Reference	Patients (*n*)	HIV-positive (%)	Virology result							
			>1 virus	Influenza virus	Adenovirus	Rhinovirus	PIV1-3	RSV	hMPV	Other
Joosting et al., 1979^a^ [44]	1012	NS	477 (47.1%)	Any: 384 (37.9%) A: 287 (28.4%) B: 87 (8.6%) C: 10(1.0%)	45 (4.4%)	-	17 (1.7%)	8 (0.8%)	-	Enterovirus: 1
Scott et al., 2000^b^ [8]	281	52%	16 (5.7%)	5% A: 12 (4.3%) B: 2 (0.7%)	2 (0.7%)	-	-	0	-	
Hartung et al., 2011^c^ [45]	51	94%	11 (21.6%)	A: 1	1 (9%)	4 (36%)^d^	PIV1: 1 (9%)	1 (9%)		Bocavirus: 1 (9%) Coronavirus NL63: 1 (9%); Coronavirus OC43: 1 (9%)
Feikin et al., 2012^e^ [39]	396^f^	49%	230 (58%)	Any: 37 (9%) A: 29 (7%) B: 8 (2%)	29 (7%)	39 (35%)^h^	PIV1: 2 (0.5%) PIV2: 6 (2.0%) PIV3: 14(4.0%)	33 (8%)	10 (3%)	
Pretorius et al., 2012^i^ [41]	4006^g^	NS	5–24 years: 51% 25–44 years: 33.8% 45–64 years: 28.7% >65 years: 24.2%	Any: 343 (8.6%) A(H3N2): 102 (2.5%) A(H1N1): 104 (2.6%) B: 137 (3.4%)	251 (6.3%)	653 (16%)	Any: 145 (3.4%) PIV1: 12 (0.3%) PIV2: 27 (0.7%) PIV3: 96 (2.4%)	134 (3.3%)	62 (1.5%)	Enterovirus: 63 (1.6%)

HIV, human immunodeficiency virus; NS, not stated; PIV1-3, parainfluenza virus type 1 to 3; RSV, respiratory syncytial virus; hMPV, human metapneumovirus.

^a^Tested for Influenza A, B & C viruses, PIV1-3, RSV, adenovirus, herpesvirus, and enterovirus.

^b^Tested for Influenza A & B viruses, adenovirus, and RSV.

^c^Tested for Influenza A & B viruses, PIV1-4, adenovirus, rhinovirus, RSV type A & B, hMPV; Coronavirus 229E & OC43, human Coronavirus NL63, bocavirus.

^d^2 were co-infected with *Pneumocystis jiroveci* pneumonia; 1 with pulmonary *Mycobacterium tuberculosis*; 1 with pulmonary Kaposi’s sarcoma.

^e^Tested for Influenza A & B viruses, PIV1-3, adenovirus, RSV, and hMPV (also rhinovirus, enterovirus and parechovirus from January 2009).

^f^Only included hospitalised patients aged >5 years in whom naso/oropharngeal specimens taken.

^g^Only included patients aged >5 years.

^h^Include enterovirus due to cross-reactivity on real-time transcriptase polymerase chain reaction (RT-PCR).

^i^Tested for Influenza A & B viruses, PIV1-3, adenovirus, RSV, hMPV, rhinovirus and enterovirus.

The percentage of patients with at least one viral pathogen ranged from 5.7% to 58% (Table 2), which is comparable to the 11–56% reported in contemporary studies in developed settings (16–21, 47). The earliest study, conducted in South African black miners hospitalised with acute respiratory disease (definition not stated), reported detection of a respiratory virus in 47% of cases using viral culture and serology (44). In particular, a very high proportion of influenza A (28%) virus was found, even though whole years of observations were included. The authors attributed the prolonged influenza epidemic in the miners to the close working conditions that facilitated transmission and high staff turnover which maintained a large pool of susceptible individuals. In contrast, a prospective hospital-based study by Scott et al. (8) only identified a viral pathogen in 5.7% of adults with hospitalised pneumonia in Kenya. However, serology was the sole viral diagnostic method used to test for a small number of viruses.

A prospective observational study in pneumonia patients admitted to a high dependency unit (HDU) of a tertiary referral hospital in Malawi found a viral pathogen in 11 of 51 (22%) patients (45) — 3 had a final diagnosis of viral pneumonitis, whereas the rest had an alternative diagnosis (pulmonary Kaposi’s sarcoma) or pathogen (e.g., *Mycobacterium tuberculosis)* that was considered the primary cause for severe respiratory presentation. The study was limited to those who could tolerate a bronchoscopy (with resultant exclusion of 39% of eligible patients), which likely introduced selection bias. Moreover, two-thirds of patients had symptom duration of greater than 3 weeks, which may have accounted for the high proportion of *Pneumocystis jiroveci* pneumonia (PCP) (27%) and *M. tuberculosis* (22%).

The latest studies are derived from epidemiological surveillance in Kenya (39) and South Africa (41). Both studies reported high rates of viral infection — in particular, high prevalence of rhinovirus, enterovirus, influenza virus and adenovirus were found (Table 2). Feikin et al. (39) also enrolled asymptomatic hospital controls, hence the authors were able to infer the absence of association with clinical illness in a number of commonly identified viruses (namely, rhinovirus, enterovirus and adenovirus). Conversely, influenza A virus and RSV were significantly associated with hospitalised ARI. Pretorius et al. (41), the largest study to date with greater than 4,000 patients, was able to report age-stratified prevalence of detected viruses. They demonstrated the highest prevalence of viruses in hospitalised severe acute respiratory infection (SARI) patients aged 5–24 years (51%), compared to older age groups (25–44 years, 34%; 45–64 years, 29%; >65 years, 24%).

Outcome data were sparse among the included studies — mortality of 10% and 22% were reported by Scott et al. (8) and Hartung et al. (45), respectively; Feikin et al. (39) found a case fatality ratio of 6% in persons aged 5 years and over with hospitalised ARI. However, none of the studies reported clinical outcome according to underlying viral or bacterial aetiology. Moreover, there were limited data on high-risk groups such as HIV-infected individuals, adults with underlying co-morbidities, and the elderly. Feikin et al. (39) described higher incidence rates of all pathogens in HIV-infected compared to HIV-uninfected adults, but this analysis included both inpatients and outpatients. Pretorius et al. (41) did not find higher prevalence of virus in adults aged 65 years and over compared to younger age groups. Although beyond the scope of this review, young children are also an important risk group. The aetiology and risk factors for hospitalised pneumonia in this group are being addressed by the Pneumonia Etiology Research for Child Health (PERCH) project, a multi-site case control study in 7 countries, 5 of which are in sub-Saharan Africa (48).

### 3.1 Multiple pathogen infection

As a result of an expanding armamentarium of diagnostic tests, viral-viral and viral-bacterial co-infections are increasingly identified. Several studies in well-resourced settings have found more severe disease and poorer outcomes in patients with polymicrobial infections (16, 17, 19, 22). However, the clinical interpretation of mixed infections is not straightforward since detection of multiple pathogens could reflect three possible scenarios: i) all detected organisms have contributed to pathogenesis; ii) >1 detected organism is an innocent bystander; iii) one organism is the predisposing factor (e.g., by damaging the respiratory epithelium) to cause pneumonia by the second organism.

Influenza virus and *S. pneumoniae* is the most studied co-infection combination; their synergistic interaction is well described (49, 50). In the African setting, the pneumococcal conjugate vaccine (PCV) has been shown to prevent 31% of viral pneumonia in South African children (51), providing epidemiological evidence of the importance of pneumococcal co-infection in viral pneumonia. More recently, a hospital-based surveillance study in South Africa described elevated blood pneumococcal load (as a marker for invasive pneumococcal pneumonia) in patients with influenza virus co-infection (52).

All but one of the included African studies described mixed infection. Scott et al. (8) reported bacterial pneumonia in 8 of 12 cases with influenza. Viral co-infections (rhinovirus [n = 3]; coronaviruses OC43 and NL63; RSV and PIV type 1 [PIV1]) with bacteria/fungus (including *Staphylocccus aureus, Klebsiella pneumoniae, P. jiroveci* and *M. tuberculosis*) were demonstrated in 7 of 51 (14%) cases of the Malawian HDU cohort (45). The recent ARI surveillance study in Kenya demonstrated mixed viral and bacterial infections in 29% of patients (39, 41). Co-infections were more common in HIV-positive than HIV-negative patients, and influenza virus was less likely to be co-infected with other viruses, compared with other viral pathogens (39). Pretorius et al. (41) reported viral co-infections in 17% of hospitalised pneumonia patients (this included children <5 years), but were unable to correlate co-infection with clinical outcome due to the low number of specific co-infection combinations. Among co-infected patients in the Kenyan and South African studies, adenovirus, rhinovirus and RSV were the most frequently detected viruses. Feikin et al. (39) found a similar prevalence of rhinovirus and adenovirus in cases and controls, implying that their identification in ARI cases may not be clinically significant. Nonetheless, the contribution of these commonly identified viruses to disease severity is often unclear from available literature.

The role of rhinovirus in pneumonia is contentious. On one hand, asymptomatic carriage is well described in immunocompetent (53) and immunocompromised patients (54). Conversely, viral shedding beyond 2 weeks of acute illness is rare, suggesting an association with clinical disease (55, 56). Furthermore, rhinovirus has been associated with hospitalised LRTI in Thai adults (57), and has also been the suspected causative organism in several outbreaks of severe respiratory diseases in nursing homes in the United States (58, 59).

In contrast, asymptomatic carriage of adenovirus (60) and RSV (61) are uncommon. Hence their presence is usually pathogenic. Adenovirus has been implicated as a cause of pneumonia in South African and Chinese adults, although neither study had a control group (44, 62).

### 3.2 Limitations of current studies

Given the wide variation in time period, study population, pneumonia definition, clinical specimens, diagnostic methods, and number of pathogens tested, it is difficult to make any firm conclusions from the handful of studies available beyond that viral pathogens are prevalent in older children and adults in sub-Saharan Africa presenting with severe ARI. Mixed infections are common. With the exception of Feikin et al. [39], the lack of control group is a major limitation, as the background prevalence of asymptomatic viral infections is undefined (see section 4.2 for the role of healthy controls).

## 4. Diagnosis of viral pneumonia

### 4.1 Evolution of viral diagnosis in acute respiratory infections

Conventional diagnostic methods, such as, viral culture and immunofluorescence microscopy of respiratory specimens or serological testing of acute and convalescent serum samples, have limited clinical applicability due to lengthy processing time and poor sensitivity (63). Nucleic acid amplification tests (NATs), such as PCR-based methods, have greatly enhanced detection of respiratory viral pathogens. They demonstrate superior sensitivity compared to conventional methods (64), are able to detect viruses that are difficult to culture (e.g., hMPV, coronaviruses NL63 and HKU1, and rhinovirus), can rapidly characterise new viral pathogens (65), and can yield results in a clinically relevant time frame. Moreover, multiplex platforms enable simultaneous detection of a large number of viral pathogens from a single specimen, thus facilitating diagnostic efficiency and cost-effectiveness (66). Studies in well-resourced settings have demonstrated higher microbial yield when conventional diagnostic techniques are augmented by NATs (16–19), which now form the backbone of respiratory viral testing in both well resourced and surveillances sites in low-income settings.

### 4.2 Challenges in interpretation of viral diagnostic results

Lower respiratory tract samples (e.g., induced sputum, bronchoalveolar lavage or transthoracic needle aspiration) provide the most sensitive specimens for determining the likely cause of pneumonia since they are taken from the site of infection (67, 68). However, they are difficult to obtain and unfeasible in most African settings. Viral diagnosis therefore mostly relies on upper respiratory specimens, but the high sensitivity of molecular tests complicates the interpretation of a positive result (69). Viruses identified from nasopharyngeal specimens can be the underlying cause of LRTI, but may also represent contamination, colonisation, post-infectious shedding, or the predisposing agent to subsequent bacterial superinfection. Thus, attributing causality to every positive PCR result may overestimate the contribution of respiratory viruses, particularly if diagnostic tests for other pathogens, such as blood culture, have comparatively low sensitivity.

Molecular results should be interpreted in the context of the patient’s clinical presentation and known epidemiology of the detected viral pathogens. Additionally, viral load quantification (70, 71) or ascertainment of background prevalence of asymptomatic nasopharyngeal viral infection in a control group (39, 48) could further clarify the pathogenic roles of detected viruses. A study design analogous to the PERCH study, whereby multiple upper and lower respiratory specimens are obtained from pneumonia cases and community controls, will allow the strength of association between a positive result and pneumonia to be established, in addition to comparison of concordance of microbial results between upper and lower respiratory specimens from the same patient (48).

### 4.3 Expansion of diagnostic capacity in Africa

Over the past decade, the emergence of the highly pathogenic avian influenza A(H5N1) virus and pandemic influenza A(H1N1) virus highlighted the need to strengthen capacity for ARI surveillance in sub-Saharan Africa (43). With financial and technical support from national and international institutions such as the World Health Organisation (WHO), Centers for Disease Control and Prevention (CDC), Institut Pasteur and the National Institute for Communicable Diseases in South Africa, a growing number of countries in the region have acquired PCR diagnostic capabilities (35, 43). At least 24 African countries now contribute specimens to WHO influenza surveillance (72). Nevertheless, the operation of a molecular diagnosis laboratory in resource-poor settings is associated with major challenges — the requirements for reliable power source; access to reagents, equipment, trained technicians and skilled maintenance; in addition to robust sample transfer and storage systems and stringent quality control, represent significant barriers to widespread implementation of PCR testing in Africa (73). For the foreseeable future, PCR diagnostics are likely to be confined to research settings and institutionally-supported surveillance programmes. However, demonstration of substantial burden of respiratory viruses in ARI may gather momentum for global health partners to expand respiratory viral diagnostic capability to other sub-Saharan countries, and convince governments of individual countries to invest in infrastructure to support PCR diagnosis as a step towards sustainability (35).

## 5. Management and prevention

### 5.1 Management

Antivirals against respiratory viruses are prohibitively expensive and largely unavailable in sub-Saharan Africa. Despite the likely prevalence of CMV pneumonitis, there are no published reports on ganciclovir use in adult CMV disease on the continent (11). With the exception of aciclovir for the treatment of HSV and VZV (74), anti-influenza agents, namely neuraminidase inhibitors (NI) (oseltamivir and zanamavir) and M2 inhibitors (amantadine and rimantadine), are the only antivirals available in a small number of African settings (15). Moreover, due to widespread resistance against seasonal influenza A(H3N2) virus and pandemic influenza A(H1N1) virus in recent years (75), M2 inhibitors are no longer recommended for the treatment or prophylaxis of influenza A virus (76).

NIs have been shown to be 68–92% effective in preventing influenza in healthy adults and children in developed settings (77). In a recent Cochrane review, oseltamivir was associated with a shortening of illness duration by 0.7 days (78). Due to the lack of diagnostic definitions, it is unclear whether NIs reduce the risk of influenza complications such as pneumonia (78), though they had no effect on rates of hospitalisation (78). Data on the impact of NIs on hospitalised or immunosuppressed patients with influenza pneumonia are scarce. Current evidence, largely derived from retrospective studies, suggests that oseltamivir accelerates viral clearance (79), reduces length of hospital stay (80) as well as lowers mortality in patients hospitalised with influenza (81, 82). Specifically in HIV-infected individuals, several studies have suggested that oseltamivir reduces severity of illness (83), but no prospective evaluation of the efficacy of NIs in HIV-infected individuals or in African settings has been undertaken.

NIs are most effective if administered within 48 hours of symptom onset (84). The lack of viral testing in ‘realtime’, broad differential diagnosis of acute respiratory presentations in high HIV prevalence settings, in addition to high cost and lack of availability, are barriers to the widespread and effective use of NIs in the region at present.

### 5.2 Prevention

With a shortage of therapeutic options, prevention and control of viral pneumonia should be focused on infection control and vaccines. Core infection control measures, such as cohorting and isolation, are impractical in many resource-poor settings. However, physical interventions such as handwashing, use of masks, gloves and aprons may be feasibly implemented to minimise risk of nosocomial spread (85, 86).

Vaccine options are limited for the prevention of respiratory viral infections — the only approved vaccine is against seasonal influenza. In well-resourced settings, annual influenza vaccination with trivalent or quadrivalent inactivated vaccines (TIV/QIV) or an intranasal live attenuated vaccine (LAV) has an established role in preventing influenza infection and its complications (13, 14). LAV is contraindicated in immunosuppressed or pregnant patients, thus unsuitable for widespread use in sub-Saharan Africa where HIV prevalence is high. Only 2 studies have evaluated efficacy or effectiveness of TIV in Africa, both from South Africa. Age-adjusted vaccine effectiveness of TIV, estimated from a surveillance programme on patients with influenza-like illness (ILI), ranged from −14% (95% CI-100% to 35%) to 67% (95% CI 12%–90%) between 2005 and 2009, though low vaccine coverage (approximately 4% among ILI cases) may have reduced the power to estimate vaccine effectiveness (87). A randomised double blind placebo-controlled trial of TIV in HIV-infected adults demonstrated vaccine efficacy of 75.5% (95% CI 9.2%–95.6%) (88), which is comparable to that in developed settings (89). However, excluded patients with underlying co-morbidities and advanced immunosuppression (antiretroviral therapy naïve with CD4 <100 cells/µL). Evaluation of TIV or QIV efficacy in this high-risk group is required.

A recent CDC survey reported availability of seasonal influenza vaccine in 14 of 31 (45%) African countries (15). However, only 2 sub-Saharan African countries (Côte d’Ivoire and Mauritius) reported to have a national public policy for influenza vaccination. Even if annual seasonal influenza vaccination became national policy, its implementation will be logistically challenging. There is currently no indigenous vaccine production in the region (72). Additionally, the vaccine donation initiative that provided 2009 pandemic influenza (H1N1) virus vaccines also highlighted problems in vaccine procurement, distribution and uptake in the region (72).

A number of vaccines against other important viral pathogens such as RSV (90) and PIV type 3 (PIV3) (91) are under development. Furthermore, given the protective effect of pneumococcal vaccination on viral pneumonia in children (51), the current PCV roll-out in children in a number of African countries may result in an indirect benefit on adult viral pneumonia (92).

## 6. Conclusion

Increasing deployment of sensitive molecular diagnostic assays has improved our ability to characterise the contribution of viruses to adult pneumonia. Viruses commonly identified in CAP in the Western setting-namely rhinovirus, influenza virus, RSV and PIV — are prevalent in adults and older children presenting with hospitalised pneumonia in sub-Saharan Africa. Novel viruses such as hMPV and human bocavirus are increasingly identified. This review highlights the scarcity of literature on viral aetiology in adult CAP, especially in at-risk groups such as HIV-infected individuals, though data are beginning to emerge from SARI surveillance and prospective observational studies.

Vaccine and treatment options for viral respiratory pathogens remain limited and out of reach for most of sub-Saharan Africa, and evidence for their use in this setting is lacking. Nonetheless, ongoing epidemiological surveillance is important to evaluate temporal variations of circulating viruses and anticipate future outbreaks and pandemics. Better characterisation of the burden and spectrum of viruses that contribute to adult CAP in the region will also enable more informed interpretation of positive molecular results, aid decisions on important pathogens to include in respiratory disease surveillance programmes, as well as the most pathogenic viruses to target for vaccine and antiviral development. Furthermore, establishing a substantial burden of virus-associated severe respiratory infection (in particular influenza) may persuade local policy makers to consider targeted immunisation programmes — annual influenza vaccination in at-risk adults and PCV in children could potentially prevent a substantial proportion of viral pneumonia in adults.
